# Purification Behavior of Zn(II) in Water by Magnesium Hydroxyapatite: Surface Complexation, and Dissolution–Precipitation

**DOI:** 10.3390/ijerph17113804

**Published:** 2020-05-27

**Authors:** Nan Mo, Zongqiang Zhu, Yinian Zhu, Yang Liu, Xingxing Wang, Hongqu Yang, Ningning Zhao

**Affiliations:** 1 Collaborative Innovation Center for Water Pollution Control and Water Safety in Karst Area, Guilin University of Technology, Guilin 541004, China; monan998@163.com (N.M.); liuyang0908123@163.com (Y.L.); wangxingxing026@163.com (X.W.); hongquyang@163.com (H.Y.); 18835175975@163.com (N.Z.); 2 Guangxi Key Laboratory of Environmental Pollution Control Theory and Technology, Guilin University of Technology, Guilin 541004, China; 3 Research Center for Eco-Environmental Sciences, Chinese Academy of Sciences, Beijing 100085, China

**Keywords:** magnesium calcium hydroxyapatite, characterization, Zn(II), removal performance, adsorption mechanism

## Abstract

As an innovative and economical material, hydroxyapatite does little harm to the environment. In this study, a magnesium hydroxyapatite (Mg-HAP) adsorbent was prepared by doping magnesium. Magnesium doping can increase the hydroxyl groups on the surface of Mg-HAP to form more adsorption sites and improve the removal effect of the heavy metal Zn(II) in water. This study was implemented to survey the effect of different sorption elements, including the liquor initial pH, initial concentration, dose of adsorbents, and other factors, on the adsorption effect. The outcomes show that the sorption effect was best at the time that the liquor was weakly acidic (pH = 6); At a pH of 6, the temperature of 25 °C when the optimal dosage of adsorbent is 0.25 g, the maximum adsorption amount is 62.11 mg/g. Through data fitting, the adsorption process can be accurately described as a pseudo-second-order dynamics model and the Langmuir isotherm equation. According to the thermodynamic analysis, the sorption of zinc ions by Mg-HAP belongs to the process of spontaneous endothermic and entropy increase, and the increase of temperature was conducive to adsorption. Material characterization and analysis indicate that surface complexation and dissolution-precipitation was the main mechanism for adsorption of Zn(II).

## 1. Introduction

Zinc is a universal contaminant, and it has caused widespread concern in society from the perspectives of environmental impact and human health hazards [1]. These problems are mainly the result of the irregular discharge of aqueous solution and waste residue in mining, smelting, electroplating, and other industries, such that heavy metal elements such as Zn(II) enter the environment and cause environmental pollution [2]. Zinc is very significant for human health, for example, zinc can improve people’s sleep quality [3] and serve as a marker for early prediction of prostate cancer [4], so it is an indispensable element for the human body. The World Health Organization does not provide health-based guidelines for zinc in drinking water. However, drinking water containing more than 3 mg/L of zinc may not be accepted by humans [5]. Zinc has a variety of forms, which cannot be degraded by biological processes. Instead, it is transformed between existing forms, and attached to the transmission and enrichment of the food chain, which has a tremendous impact on human health [6]. Therefore, the removal of zinc from water is of great significance to humans. There are many technical means for eliminating Zn(II), including electro-flocculation [7], chemical precipitation, ion exchange, membrane separation, nano-filtration, biological isolation, and microbial transformation [8]. On the one hand, sorption is an extraordinarily effective technique; on the other, the advantages of less investment, simplicity of design, and ease of operation for dealing with zinc-containing aqueous liquor are clear [9].

Hydroxyapatite (HAP) is an important phosphorus-containing mineral in nature and an important constituent of human bones and teeth [10]. HAP is widely used in soil remediation, water pollution, medicine, biology. It results in little environmental pollution and is considered as an environmentally friendly material. Due to its special crystal structure, HAP can adsorb heavy metal ions and some organic pollutants. However, because its adsorption effect is slow, it is not widely used. Gayathri et al. [11] argued that the incorporation of Mg into HAP can increase the biological activity of the implant and improve the effect of pesticides; Gong et al. [12] concluded that Mg-HAP can be used as an important material for bone regeneration in medicine; Chen et al. [13] argued that the use of SO_4_^2−^ loaded on HAP can increase the adsorption capacity for F^−^. Nie et al. [14] showed that the modification of HAP with Al can increase the adsorption sites on its surface and improve the removal effect of fluoride ions. Leyva et al. [15] showed that the removal rate of antimony by HAP was more than 95%. Wang et al. [16] concluded that the adsorption properties of HAP/bio-carbon materials were significant and had strong separation ability, and the removal ability of Zn^2+^ reached 90.7%. Therefore, by doping certain ions, the crystal state of HAP can be changed or even crystallized, the adsorption effect can be enhanced, and the adsorption capacity can be enhanced.

In this study, Mg-HAP was obtained by doping Mg^2+^, and the sorption effect of the heavy-metal Zn(II) was investigated. By discussing the function of different elements (for instance, initial concentration, pH, sorption dose, and temperature) and analyzing kinetics, we fully explain the sorption effect of Mg-HAP on zinc.

## 2. Materials and Methods

### 2.1. Preparation

The concentration of 0.2 mol/L solution was prepared by using magnesium nitrate hexahydrate, calcium nitrate tetrahydrate, and diammonium hydrogen phosphate, and the concentration of 500 mg/L zinc liquor was prepared by using zinc nitrate hexahydrate as the reserve solution, which was diluted appropriately, as required in the subsequent experiments.

The prepared magnesium nitrate hexahydrate, calcium nitrate tetrahydrate, and diammonium hydrogen phosphate aquas were mixed based on the molar ratios (x = Mg/(Ca + Mg)) of 0, 0.05, 0.10, 0.15, 0.20, 0.25, 0.30, 0.35, 0.40, 0.45, 0.50, 0.55, 0.60, 0.65, 0.70, 0.75, 0.80, 0.85, 0.90, 0.95, and 1.0, respectively. Briefly, 500 mL of a mixed solution of magnesium nitrate hexahydrate and calcium nitrate tetrahydratewas taken according to the molar ratio; then, according to the molar ratio (Ca + Mg)/P = 1.67, a diammonium hydrogen phosphate solution was added to mix evenly, and ammonia was simultaneously added to adjust the pH to about 10.5. After the aqua was agitated for about 30 min, it was warmed to 50 °C in a water bath for about 120 h. After reaching the reaction time, the polyethylene bottles were removed from the water bath and cooled to room temperature. Then, the solid-phase precipitate was centrifuged three times (4000 r/min, 5 min), dried at 80 °C for about 24 h, and ground to acquire magnesium calcium hydroxyapatite (Mg-HAP) series.

### 2.2. Characterization

The shape characteristics were acquired using a scanning electron microscope (SEM, Jeol JSM-6380LV, Japan Electron Optics Ltd., Tokyo, Japan), and the surface chemical composition was measured using an energy dispersive spectrum (EDS; IE350, Oxford Instruments, Oxford, UK); a powder X-ray diffractometer (XRD; X’Pert PRO, PANalytical B.V., Almelo, The Netherlands) was used to record the material before and after adsorption Zn(II); a Fourier transform infrared spectrometer (FTIR; Nicolet Nexus 470, Madison, WI, USA) was used to determine sample structure and chemical functional groups.

### 2.3. Adsorption Experiments

The Zn(II) aqueous liquor was prepared by analyzed pure zinc nitrate hexahydrate, nitric acid, and ultrapure water. Then, 0.25 g of Mg-HAP adsorbent was accurately weighed and mixed with 50 mL of a certain concentration of Zn(II)-containing aqueous liquor in a 100-mL centrifuge tube. It was placed in a water bath thermostatic shaker at 25 °C and 150 rpm. After reaction equilibrium, it was filtered through a 0.22 μm microporous membrane. The remaining concentration of Zn(II) was detected by an inductively coupled plasma-optical emission spectrometer (Optima 7000DV, Perkin-Elmer Inc., Waltham, MA, USA). After sampling, the solid-liquid separation was carried out, and the solid phase material was rinsed three times, then dried in a blast drying chest at 50 °C. The solid was characterized after dried in a blast drying oven at 50 °C.

Then, 0.25 g of Mg-HAP adsorbent was weighed at 25 °C, and 50 mL of zinc simulated aqueous solution at the initial concentrations (10, 20, and 50 mg/g) was added to a 100-mL centrifuge tube. The influence of pH was investigated under conditions of various pHs (1, 2, 3, 4, 5, 6, 7, and 8). The influence of initial concentration and adsorption isotherms were observed at different temperatures (25, 35, and 45 °C), pH 6, and various initial concentrations of Zn(II) liquor (100, 200, 300, 400, 500, 600, 700, 800, 900, and 1000 mg/L). The effect of sorbent dose was examined by adding various amounts (0.05, 0.10, 0.15, 0.20, 0.25, 0.30, 0.35, 0.40, 0.45, and 0.50 g) of Mg-HAP into the solution at pH 6 and Zn(II) initial concentrations of 10, 20, and 50 mg/L. In order to examine the influence of kinetics, 0.25 g of Mg-HAP and 50 mL of Zn(II)-containing solutions with different concentrations (75, 100, and 150 mg/L) were added to a 100-mL centrifuge tube, and the liquor sampling was then removed from various centrifuge tubes at time intervals of 5, 10, 15, 20, 30, 45, 60, 120, 180, 240, 300, 360, 420, 480, 540, 750, 1080, and 1440 min, respectively.

## 3. Results

### 3.1. Characterization of the Sorbent

#### 3.1.1. Powder X-ray Diffraction

**Figure 1:**
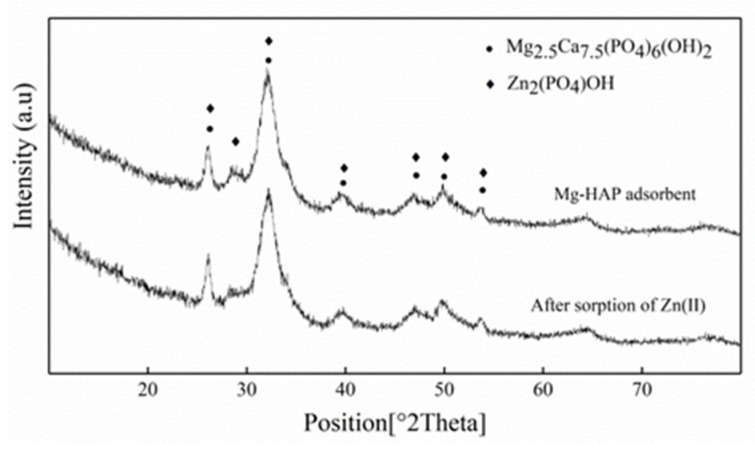
X-ray diffraction (XRD) patterns of magnesium hydroxyapatite (Mg-HAP) before and after Zn(II) sorption. Initial pH = 6.0; adsorbent dose = 0.25 g/50 mL, 25 °C.

In order to observe the situation before and after Mg-HAP adsorption, the powder X-ray diffractometer (XRD) was used to characterize the adsorbent, with a scanning range of 10°~80°. The diffractograms obtained before the sorption of Zn(II) by the Mg-HAP was consistent with HAP in terms of peak position and intensity (Figure 1). The peaks at 2θ = 26.11, 28.58, 32.21, 46.76, 49.83, and 53.78° corresponded to the (002), (120), (112), (222), (123), and (004) reflection peaks of HAP (reference code 01-084-1998). XRD analysis showed that after the adsorption of Zn(II) by Mg-HAP, no significant shift of the diffraction peak was observed. A HAP diffraction peak on the adsorbent was identified (reference code 00-001-0964). According to the ionic radius analysis of the elements, the ion radius of Ca(II) (0.99 Å) is less than that of Zn(II) (0.74 Å), which indicated that larger Ca(II) ions in HAP could be replaced by smaller Zn(II) ions [17]. At the same time, it was observed that all the peaks turned to a lower diffraction angle, which may have caused the solid solution structure to shrink. XRD analysis confirmed that the Zn_2_(PO_4_)OH solid solution was formed after Mg-HAP adsorbed Zn(II).

#### 3.1.2. Scanning Electron Microscope (SEM) and Energy Dispersive Spectrum (EDS)

**Figure 2:**
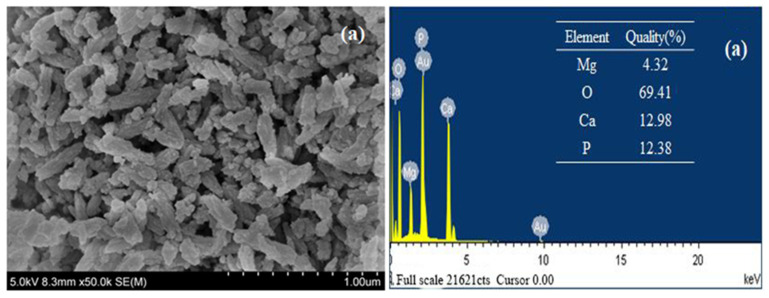

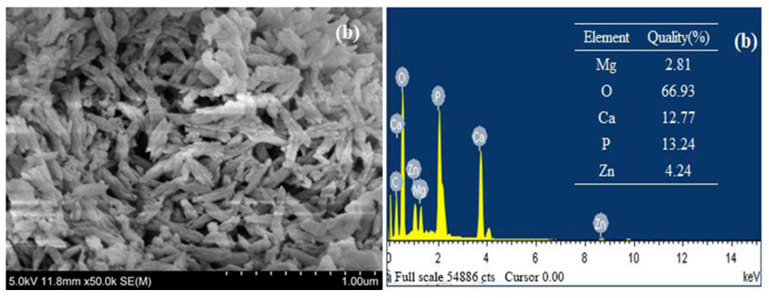
Scanning electron microscope (SEM) results for the Mg-HAP adsorbent and energy dispersive spectrum (EDS) analysis results for the Mg-HAP sorbent (**a**) before and (**b**) after Zn(II) sorption (initial concentration = 10 mg/L; initial pH = 6.0; sorbent dosage = 0.25 g/50 mL; 25 °C).

The micromorphology of Mg-HAP before and after zinc sorption can be clearly observed via SEM. When Mg-HAP was not adsorbed, its surface exhibited a rough, compact, and short-rod-like appearance (Figure 2a), and there was no change when the adsorption was completed. It was also observed that the void structure was more obvious so that Mg-HAP did not affect the crystal structure of the solid solution after adsorbing Zn(II), and this structure facilitated the adsorption of Zn(II) ions. Corresponding EDS analysis ascertained the presence of-Mg, O, Ca, P (Figure 2a) material samples. Au was introduced during the preparation, whereas Mg, O, Ca, P, and Zn (Figure 2a) elements were observed after adsorption. The elements in the HAP were present in the test results, and the Mg appeared at the same time, indicating that Mg had entered the HAP crystal lattice. According to related studies, Ca^2+^ in the HAP component can easily be displaced with some small radius ions [18]. However, the increase of the Mg-HAP-specific surface area is more conducive to the precipitation of Mg^2+^ and Ca^2+^ from the lattice of HAP [19]. In Figure 2a,b, the mass fractions of Mg and Ca decreased from 4.32% and 12.98% to 2.81% and 12.77%, respectively, and the mass fraction of Zn after the completion of adsorption was 4.24%, indicating that Zn(II) ion may exchange with Mg^2+^ and Ca^2+^ [20].

#### 3.1.3. Fourier Transform Infrared Spectrometer (FTIR)

**Figure 3:**
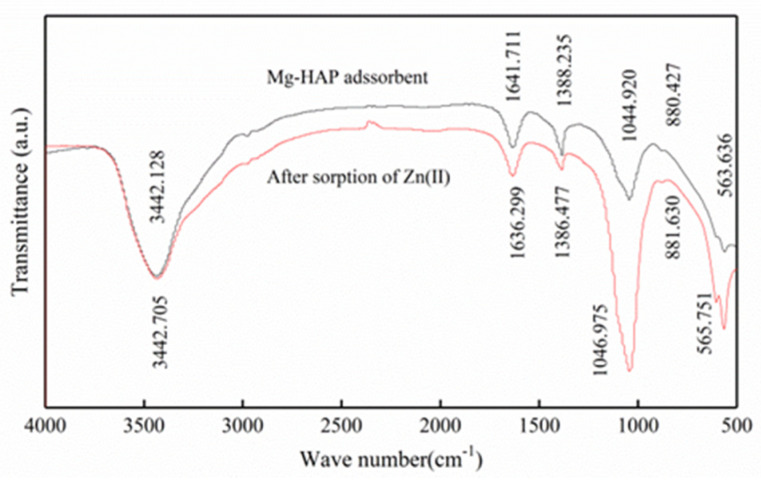
FTIR spectra of Mg-HAP before and after zinc sorption.

In order to observe the structure of Mg-HAP before and after the sorption of zinc (Figure 3), its surface features were analyzed via FTIR. FTIR analysis indicated that the P-O link bending mode of Mg-Hap was at 563.636 and 565.751 cm^−1^ [11]. The stretch mode of a phosphate group was at 1044.920, 1046.975 cm^−1^ [21]. The strip at 880.427 cm^−1^ was considered an asymmetric telescopic motion associated with the P-O bond [11]. Therefore, these analyses indicate the presence of a PO_4_^3−^ group. However, the band at 1641.711, 1636.299 cm^−1^ may have been an absorbed water molecule [22] and had a tensile mode of O-H bond at 3442.128, 3442.705 cm^−1^. Therefore, the tensile strength exhibited at 1044.920 and 1046.975 cm^−1^ may have been an increase in the strength of the PO_4_^3−^ absorption summit, indicating that PO_4_^3−^ participated in the chemical reaction during the adsorption process, possibly forming a phosphate complex with PO_4_^3−^ and Zn^2+^.

### 3.2. Influences of Adsorption Conditions

#### 3.2.1. Influence of Initial Solution pH

**Figure 4:**
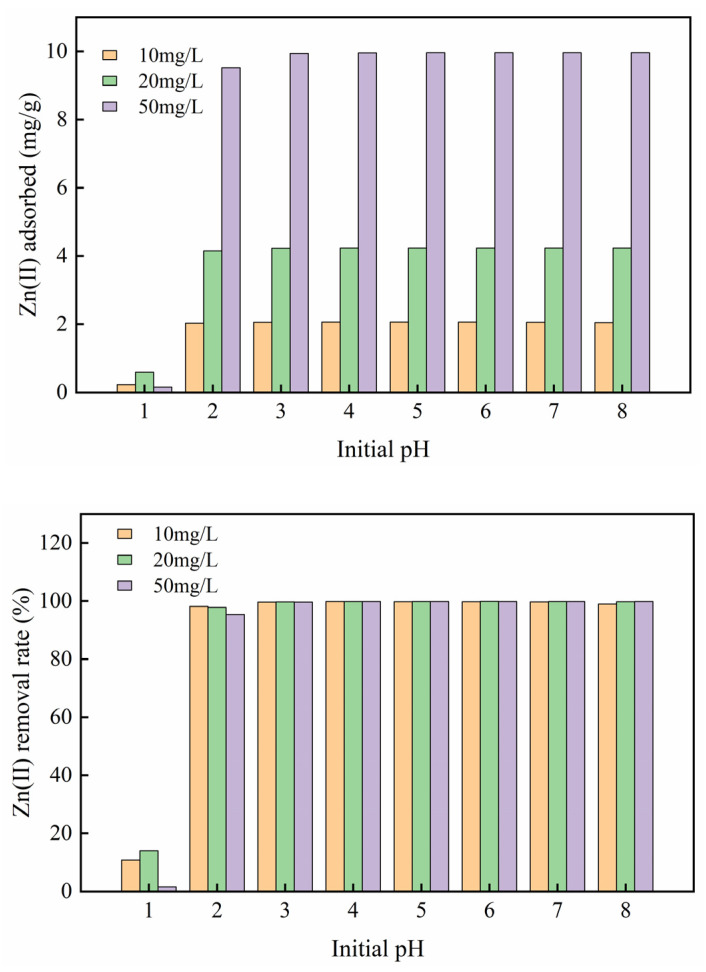
Influence of initial pH on zinc sorption on the Mg-HAP sorbent (initial concentration = 10, 20, and 50 mg/L; sorbent dosage = 0.25 g/50 mL; 25 °C).

The pH of the solution is a significant parameter [23]. Zn(II) ions can form a hydroxide precipitate at the alkaline liquor. As a result, the pH gradient was selected to be 1.0−8.0. The adsorption result of Mg-HAP adsorbent on Zn(II) is shown in Figure 4. The elimination effect and sorption capacity of Mg-HAP for three different concentrations of Zn(II) solution exhibited similar trends. The results showed that Mg-HAP did not achieve the removal effect on Zn(II) at pH = 1. When the pH is 1.0~2.0, the sorption amount and elimination rate of the solution increased rapidly, which indicated that there were abundant sites closely related to cations on the skin layer of the sorbent and that it was conducive to sorption [24]. In particular, the adsorption amount at a concentration of 50 mg/g is 2−5 times higher than 10 and 20 mg/g. When the pH > 4.0, the removal rate of the aqueous solution could be maintained at about 99% and was relatively stable. This phenomenon occurred mainly because under the condition of a strong acid, H^+^ will compete with the Zn(II) in the solution for adsorption position, and it is easy to occupy the adsorption site, at which time Zn(II) can only remain in the solution. Under weak acid conditions, more adsorption sites were provided to Zn(II) at this time, which raised the effective contact area and the amount of zinc adsorption. The adsorption effect was the best at pH = 6. At this time, the removal rates of the three different concentrations of the solution were 99.84%, 99.87%, and 99.86%, respectively, and the adsorption amounts were 2.061, 4.236, and 9.958 mg/g, respectively.

#### 3.2.2. Influence of Initial Concentration and Temperature

**Figure 5:**
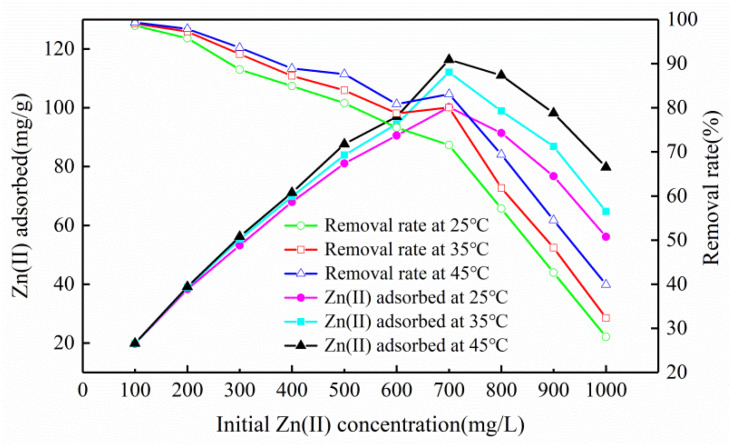
Influence of initial concentration and temperature on zinc sorption on the Mg-HAP sorbent (initial pH = 6; sorbent dosage = 0.25 g/50 mL).

With a constant increase of zinc concentration, the slope of the curve formed by the adsorption amount was first positive and then negative, indicating that the adsorption amount first increased and then decreased (Figure 5). However, the slope of the removal rate was negative, indicating that it gradually decreased (Figure 5). This phenomenon occurred because the adsorbent surface sites were abundant at the initial stage, providing enough channels to adsorb a great quantity of Zn(II) ions [25]. When the concentration reached a certain level, the surface of the adsorbent was fully occupied by heavy metal ions, the adsorption amount reached a saturation state, and the adsorption amount and removal rate were reduced. At 25, 35, and 45 °C, the maximum sorption capacity of Mg-HAP for zinc was 100.20, 112.06, and 116.34 mg/g, respectively. The removal rate changed from 98.6% to 28.06%, from 99.11% to 32.36%, and from 99.37% to 39.90%. From the temperature point of view, the raised temperature led to a corresponding increase in the removal ratio and sorption amount, which may have occurred because the adsorbent surface activation and available active sites increased; thus, increasing the temperature could promote the adsorption of the aqueous solution containing Zn(II) [26].

#### 3.2.3. Influence of Sorbent Dosage

**Figure 6:**
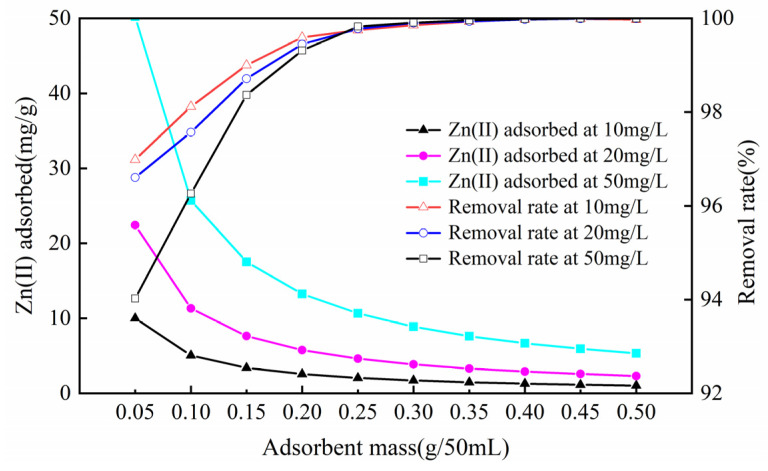
Influence of sorbent dosage on zinc sorption on the Mg-HAP sorbent (initial pH = 6; initial consistency = 10, 20, and 50 mg/L; 25 °C).

The sorbent dosage is a significant parameter that not only controls the optimal sorption capacity but also more accurately expresses the sorption effect. As the dosage of sorbent increased by degrees, the removal rates of three different concentrations of Zn(II) solution first increased and then stabilized (Figure 6). The quality of sorbent used in the experiment increased from 0.05 g to 0.50 g, and the elimination rate increased from 96.98%, 96.60%, and 94.02% to 99.97%, 99.99%, and 99.99%, respectively. The adsorption amount generally showed a downward trend, and the declining trend was faster at 0.05~0.15 g; The trend was relatively flat at 0.15~0.50 g. The adsorption amounts decreased from 10.009, 23.422, and 50.227 mg/g to 1.031, 2.320, and 5.341 mg/g, respectively. This was mainly due to the augmented dose of the sorbent and the enlarged surface area of the sorption, which raised the sorption position for the zinc ions and increased the adsorption capacity for metal ions. When the dosage increases to a certain point, more adsorption sites will be added, and the adsorption residual phenomenon will occur, thus reducing the adsorption capacity [27]. The experimental process used a sorbent dose of 0.25 g/50 mL; the removal rates of 10, 20, and 50 mg/g Zn(II)-containing aqueous solutions were 99.75%, 99.78%, and 99.82%, respectively. Therefore, considering factors such as adsorption and economic effect, the preferred dosage was 0.25 g/50 mL.

#### 3.2.4. Influence of Contact Time

**Figure 7:**
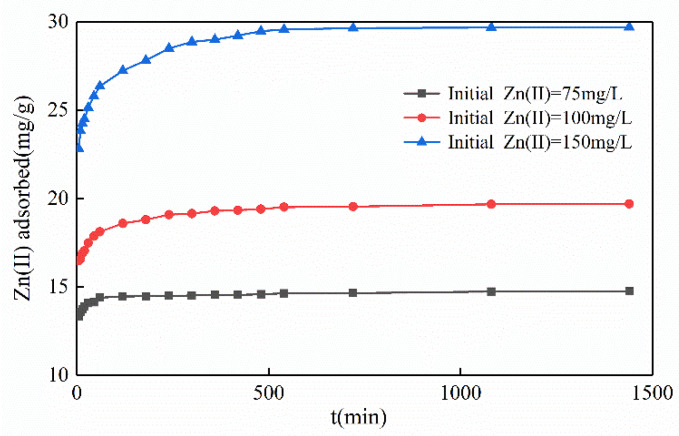
Influence of contact time on Zn(II) sorption on the Mg-HAP sorbent (initial pH = 6; sorbent dosage = 0.25 g/50 mL; 25 °C).

Contact time is a major parameter. The reaction was divided into a rapid adsorption phase and an adsorption equilibrium phase (Figure 7). In the rapid phase, for the adsorption with the initial Zn(II) concentration of 75, 100, and 150 mg/L increased continuously within 120, 340, and 560 min, respectively. This phenomenon indicated that the Mg-HAP sorbent provided abundant sorption sites for Zn(II) at the beginning, which could rapidly replace the position of H^+^ in HPO_4_^2−^ to form surface complexes. In the equilibrium phase, the adsorption basically reached the saturation state and replaced the process of ion exchange between some calcium ions and magnesium ions, the scale of sorption sites lessened and the activity decreased, and the activity ultimately reduced the reaction rate [28]. However, at pH = 6, the initial concentration of zinc was 75 mg/L, the Mg-HAP could withstand the ultimate adsorption capacity of Zn(II) within 120 min, the sorption efficiency reached 0.1205 mg/(g·min), and the total amount of adsorbed Zn(II) reached 97.28%. Similarly, when the Zn(II)-containing liquor was 100 and 150 mg/L, respectively, over 95% of zinc was adsorbed in 300 min, and the sorption efficiency reached 0.0638 and 0.0962 mg/(g·min).

### 3.3. Sorption Dynamics and Isotherm

#### 3.3.1. Sorption Dynamics

For the sorption dynamics characteristic curve, the pseudo-first-order dynamics model (Figure S1), the pseudo-second-order dynamics model, the Morrist particle intimal diffusion model (Figure S2), and the Elovich equation (Figure S3) were used for fitting calculations. The four sorption dynamics model and fitting parameters of Mg-HAP on zinc as follows [29]:

According to the results in Table 1, the dynamics of Mg-HAP sorption of zinc are more suitable to the pseudo-second-order dynamics. The dynamics expression is

(1) $$t/q_{t}=1/\left(K_{2}q_{e}^{2}\right)+t/q_{e}$$

where *q_t_*, *q_e_* represent the sorption capacity produced at t and equilibrium, respectively (mg/g), and *K*_2_ is a pseudo-second-order constant (g/(mg·min)).

In order to calculation sorption rate(h) (mg/(g·min)), the expression is

(2) $$h=K_{2}q_{e}^{2}$$

**Figure 8:**
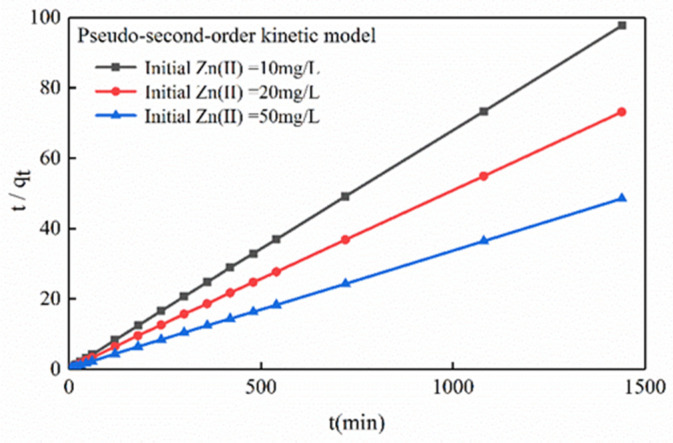
Dynamics of zinc sorption onto the Mg-HAP sorbent (initial pH = 6; initial concentration = 10, 20, and 50 mg/L; sorbent dosage = 0.25 g/50 mL; 25 °C).

The experimental results showed that the slopes and intercepts of the curves *t*/*q_t_* and *t* were used to represent the equilibrium sorption capacity (*q_e_*) and the pseudo-second-order constant (*K*_2_) (Figure 8).

The fitting correlation coefficients *R*^2^ at an initial experiment concentration of 10, 20, and 50 mg/L were 1.0000, 1.0000, and 0.9999, respectively, reaching a significant correlation. Therefore, the pseudo-second-order dynamics were better able to tally with the sorption process of zinc by Mg-HAP adsorbent. Table 2 shows that the *K*_2_ and h figures were 0.1822−0.2770 and 39.6399−206.0935 g/(mg·min), respectively. The calculated theoretical equilibrium sorption amounts were 14.75, 19.72, and 29.85 mg/g, respectively, which tallied with the practical outcomes of 14.74, 19.69, and 29.68 mg/g. Therefore, it can be speculated that the process of Mg-HAP sorption of zinc is mainly based on the effect of chemical reaction [30]. The fitting *R*^2^ value of the Elovich equation (Table S1) was highly consistent with the pseudo-second-order kinetic fitting *R*^2^ value, which further indicates that there may have been an ion exchange between Mg-HAP and Zn(II).

#### 3.3.2. Adsorption Isotherm

In order to investigate the sorption performance of Mg-HAP, it was described by the sorption isotherms of Langmuir and Freundlich [31] (Figure S4 and Table S2). The equations were expressed as follows:

(3) $$C_{e}/q_{e}=1/\left(q_{\max}\times K_{L}\right)+C_{e}/q_{m}$$

(4) $$\ln q_{e}=\ln k_{F}+1/n\;\ln C_{e}$$

where *q*_max_ represents theoretical maximum sorption capacity (mg/g); *C_e_* represents equilibrium concentration (mg/L); *K_L_* represents the Langmuir constant (L/mg); *K_F_* represents the Freundlich constant.

**Figure 9:**
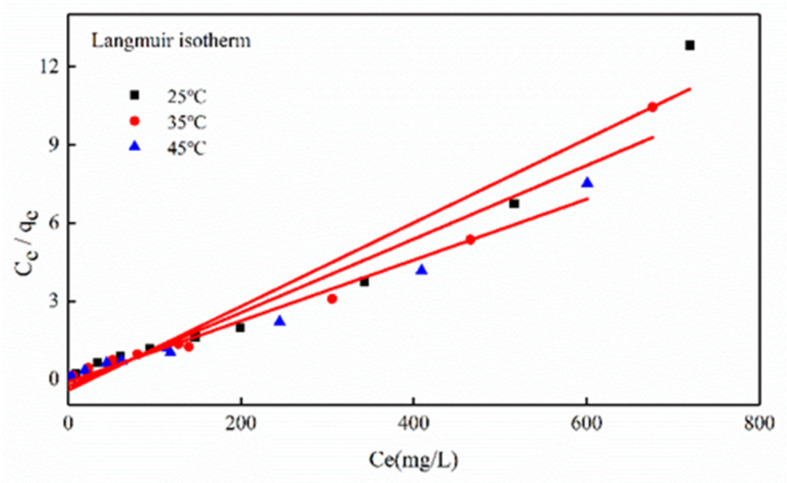
Adsorption isotherm for Zn(II) sorption onto the Mg-HAP at 25, 35, and 45 °C (initial pH = 6; initial concentration = 10, 20, and 50 mg/L; sorbent dosage = 0.25 g/50 mL).

Figure 9 shows the data-fitting results for *C_e_*/*q_e_* vs. *C_e_*, namely, the resulting linear plot slope and intercept could estimate the *q_m_* and *k_L_* constants (Table 3). Moreover, the sorption of Zn(II) by Mg-HAP adsorbent followed the Langmuir isotherm.

The properties of the Langmuir isotherm were described using a dimensionless constant (*R_L_*). The equation is

(5) $$R_{L}=1/\left(1+K_{L}C_{0}\right)$$

where *C*_0_ is the initial Zn(II) concentration (mg/L).

From the analysis results, it was clear that the adsorption of Zn(II) by Mg-HAP can be more accurately described by the Langmuir isotherm model. However, at 25, 35, and 45 °C, the resulting *R*^2^ values were 0.9677, 0.9608, and 0.9821, according to data analysis (Table 3). *R*^2^ > 0.95 indicated a uniform feature in the skin layer of Mg-HAP, and it resulted in the same activation energy on the skin layer of each sorbent molecule [24]. The experimental data for Zn(II) adsorption onto Mg-HAP can be accurately described by the Langmuir model, further illustrating dominated by monolayer sorption [26]. The adsorption property was further described by the separation factor *R_L_*. If 0 < *R_L_* < 1, the sorption process was favorable; *R_L_* > 1, the sorption process was unfavorable; *R_L_* = 1, the sorption process was reversible; *R_L_* = 0, the sorption process was irreversible [32]. Experimental results confirmed that 0 < *R_L_* <1, suggesting the favorable sorption of Zn(II) in aqueous solution. Moreover, the data analysis showed that the temperature and RL values related to a negative correlation, further confirming that increase temperature favored the sorption process.

### 3.4. Adsorption Thermodynamics

Temperature is a significant parameter for adsorption. Therefore, the effects of Gibbs free energy (Δ*G^θ^*), enthalpy (Δ*H^θ^*), and entropy (Δ*S^θ^*) on Mg-HAP sorption were further investigated at 25, 35, and 45 °C. The thermodynamics expression is

(6) $$\Delta G^{\theta }=-RT/\ln k_{c}$$

(7) $$\ln k_{c}=\Delta S^{\theta }/R-1/RT$$

where *R* represents the gas constant (8.314 J/(mol·K)); *K_c_* represents the ratio of the concentration at balance and remaining concentration; Δ*H^θ^* and Δ*S^θ^* are calculated from the linear slope and intercept of ln*k_c_* vs. 1/*T*.

The results are shown in Table 4. At three different temperatures, the Gibbs free energy calculations were below 0, indicating that the sorption of Zn(II) in water by Mg-HAP was spontaneous. When the temperature was raised, the value of Δ*G^θ^* gradually decreased, indicating that the extent of spontaneous reaction was positively correlated with temperature. The enthalpy (Δ*H^θ^*) > 0, which well explained the sorption of zinc in aqueous liquor by Mg-HAP, was an endothermic process. Raising the temperature favored adsorption reaction, which was consistent with the conclusions obtained using the Langmuir isotherm model. The entropy (Δ*S^θ^*) > 0 indicated that the order of the Mg-HAP adsorption system increased and that the adsorption was relatively stable.

### 3.5. Adsorption Mechanisms

The adsorption mechanism is a significant part of the adsorption process, but it is relatively complicated. Some researchers have maintained that the mechanism of zinc removal is mainly ion exchange. Calcium ions are dissolved in the HAP lattice, and the radius of calcium ions is larger than that of zinc ions, which are easily replaced by zinc ions to form new zinc-containing compounds. Sheha et al. [33] argued that the mechanism of zinc removal is not only ion exchange but possibly precipitation as well. Mavropoulos et al. [34] demonstrated that the mechanism of HAP removal of heavy metals includes not only ion exchange and dissolution-precipitation but also surface complexation.

**Figure 10:**
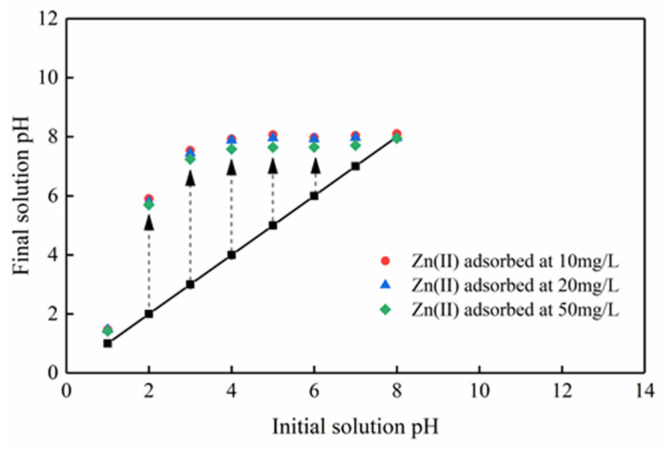
Variation of the solution pH during Zn(II) adsorption onto the Mg-HAP adsorbent.

**Figure 11:**
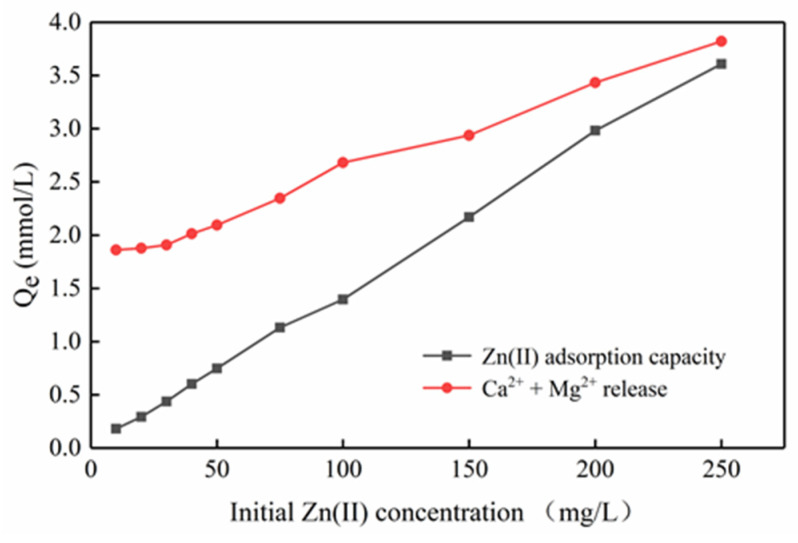
Zn(II) adsorption and Ca^2+^, Mg^2+^ release under different Zn(II) concentrations.

It is generally believed that pH is significant for the adsorption mechanism. Under different initial zinc concentration gradients, the final equilibrium pH decreases as the initial concentration increases (Figure 10). The decrease in pH indicates that due to the exchange with zinc ions, H^+^ on the surface of Mg-HAP is released into the solution, such that the formation of complexes on the surface of the adsorbent play an important role in removing zinc ions. Moreover, Figure 11 shows that the adsorption amount of zinc ions is not equal to the release amount of calcium and magnesium ions at different concentrations, confirming that there may be multiple adsorption mechanisms in the process of zinc ion adsorption. Therefore, the mechanism by which Mg-HAP adsorbent removes Zn(II) from aqueous solution may be ion exchange, surface complexation, or dissolution–precipitation. In the early stages of the kinetic reaction, due to the rapid complexation reaction of Zn(II) ions on the Mg-HAP adsorbent particles [23], the final equilibrium pH in the solution decreases, which may cause H^+^ to be located at the ≡POH site on the surface of HAP. The specific surface complexation mechanism is as follows:

(8) $$\mathrm{Mg-HAP-OH}+{\mathrm{Zn}}_{\mathrm{aq}}^{2+}\Leftrightarrow {\mathrm{Mg-HAP-O-Zn}}^{+}+H_{\mathrm{aq}}^{+}$$

(9) $$2\mathrm{Mg-HAP-OH}+{\mathrm{Zn}}_{\mathrm{aq}}^{2+}\Leftrightarrow {\left(\mathrm{Mg-HAP-O}\right)}_{2}\mathrm{Zn}+2H_{\mathrm{aq}}^{+}$$

XRD and EDS results also confirmed the surface complexation mechanism. However, the Mg-HAP adsorbent can be partially dissolved under the condition of H^+^ released by complexation, and its dissolution formula is

(10) $${\mathrm{Ca}}_{\left(10-x\right)}{\mathrm{Mg}}_{x}{\left({\mathrm{PO}}_{4}\right)}_{6}{\left(\mathrm{OH}\right)}_{2}+2H^{+}\to (10-x){\mathrm{Ca}}^{2+}+x{\mathrm{Mg}}^{2+}+6{\mathrm{PO}}_{4}^{3-}+2H_{2}O$$

The existence of Zn(II) ions at different pH conditions is shown in Figure S5. At pH < 6, the Zn^2+^ ion appears in the aqueous liquor. At a pH of 8–10, it is mainly Zn(OH)^+^ ions. At a pH of 8, Zn(OH)_2_ begins to sediment and predominates at a pH of 10. When pH > 11, the solution mainly consists of Zn(OH)_3_^−^ and Zn(OH)_4_^2−^. The types of Zn(II) ions obtained indicate that the final pH of the solution after the adsorption of zinc ions is 6–8 at an initial pH of 1.0–8.0. Phosphate groups dissolved under complexation may chemically react with the zinc ions in the solution to form hopeite.

(11) $$2{\mathrm{Zn}}^{2+}+{\mathrm{PO}}_{4}^{3-}+{\mathrm{OH}}^{-}={\mathrm{Zn}}_{2}({\mathrm{PO}}_{4})\mathrm{OH}$$

## 4. Conclusions

The surface complexation and dissolution–precipitation were the main mechanisms processed for the adsorption of Zn(II). Due to the complex reaction between Mg-HAP and zinc, the H^+^ located on the surface of Mg-HAP were released into the solution, and the Mg-HAP in the solution could be dissolved under the action of H^+^, and the remaining zinc will be converted to hopeite. The magnesium-doped HAP can increase the hydroxyl groups on the surface of Mg-HAP to form more adsorption sites and can complex with zinc in the solution to improve the removal effect of heavy metal Zn(II) in water. At a pH of 6 and a temperature of 25 °C, when the optimal dosage of adsorbent is 0.25 g, the maximum adsorption amount is 62.11 mg/g. The adsorption of Zn(II) by Mg-HAP conforms to the pseudo-second-order kinetic model and the Langmuir model, indicating that the process of zinc adsorption is mainly chemical adsorption and monomolecular adsorption. According to the analysis of adsorption thermodynamics, the sorption of Zn(II) belongs to the spontaneous and entropy-increasing endothermic process, and the elevated temperature can promote the sorption effect.

## Figures and Tables

**Table 1:** Sorption dynamics model and fitting parameters of Mg-HAP on zinc.

Model	Initial Concentration (mg/L)	Equation	*q_e_* (mg/g)	*R* ^2^
Pseudo-First-Order Dynamics	10	ln(*q_e_* − *q_t_*) = −0.0064*t* + 0.2832	1.3274	0.8729
	20	ln(*q_e_* − *q_t_*) = −0.0068*t* + 1.3503	3.8586	0.8695
	50	ln(*q_e_* − *q_t_*) = −0.0017*t* + 1.7492	5.7500	0.8688
Pseudo-Second-Order Dynamics	10	*t*/*q* = 0.0678*t* + 0.1822	14.75	1.0000
	20	*t*/*q* = 0.0507*t* + 0.2270	19.72	1.0000
	50	*t*/*q* = 0.0335*t* + 0.2313	29.85	0.9999
Morrist Particle Intimal Diffusion	10	*q_t_* = 0.0328*t*^1/2^ + 13.807	-	0.6803
	20	*q_t_* = 0.0953*t*^1/2^ + 17.027	-	0.7976
	50	*q_t_* = 0.2357*t*^1/2^ + 23.888	-	0.7883
Elovich Equation	10	*q_t_* = 0.2348ln*t* + 13.165	-	0.9249
	20	*q_t_* = 0.6561ln*t* + 15.292	-	0.9769
	50	*q_t_* = 1.3631 ln*t* + 20.677	-	0.9783

**Table 2:** Dynamics parameters for zinc sorption on the Mg-HAP sorbent.

Pseudo-Second-Order Constants				
Initial Zn(II) Concentration (mg/L)	*k*_2_ (g/(mg·min))	*h* (mg/(g·min))	*q_e_* (mg/g)	*R* ^2^
10	0.1822	39.6399	14.75	1.0000
20	0.2770	107.7193	19.72	1.0000
50	0.2313	206.0935	29.85	0.9999

**Table 3:** Dynamics parameters for zinc sorption on the Mg-HAP sorbent.

Langmuir Constants				
Temperature (°C)	*q_m_* (mg/g)	*K_L_* (L/mg)	*R_L_*	*R* ^2^
25	62.11	0.0378	0.0065−0.1634	0.9677
35	70.92	0.0520	0.0047−0.1241	0.9608
45	85.47	0.1175	0.0039−0.1047	0.9821

**Table 4:** Thermodynamic parameters after Mg-HAP sorption of zinc.

*T* (k)	Δ*G^θ^* (KJ/mol)	Δ*H^θ^* (KJ/mol)	Δ*S^θ^* (KJ/(mol·K))
298	−10.399	20.2018	0.1028
308	−11.573		
318	−12.456		

## Supplementary Materials

The following are available online at https://www.mdpi.com/1660-4601/17/11/3804/s1, Figure S1: Pseudo-first-order kinetics for Zn(II) adsorption onto the Mg-HAP adsorbent (initial pH = 6; initial concentration 10, 20, and 50 mg/L; adsorbent dose = 0.25 g/50 mL; 25 °C). Figure S2: Morrist particle intimal diffusion model for Zn(II) adsorption onto the Mg-HAP adsorbent (initial pH = 6; initial concentration 10, 20, and 50 mg/L; adsorbent dose = 0.25 g/50 mL; 25 °C). Figure S3: Elovich adsorption model for Zn(II) adsorption onto the Mg-HAP adsorbent (initial pH = 6; initial concentration 10, 20, and 50 mg/L; adsorbent dose = 0.25 g/50 mL; 25 °C). Figure S4: Freundlich isotherm for Zn(II) adsorption onto the Mg-HAP at 25, 35, and 45 °C (initial pH = 6; initial concentration 10, 20, and 50 mg/L; adsorbent dose = 0.25 g/50 mL). Figure S5: Various forms of zinc in aqueous liquor. Table S1: Kinetic parameters for the Zn(II) adsorption onto Mg-HPA adsorbent. Table S2: Freundlich isotherm parameters for the Zn(II) adsorption onto Mg-HPA adsorbent.
