# Supplementary material for: Purification Behavior of Zn(II) in Water by Magnesium Hydroxyapatite: Surface Complexation, and Dissolution–Precipitation
Source: Int J Environ Res Public Health. 2020 May 27;17(11):3804. doi: 10.3390/ijerph17113804 (PMC7312566; doi:10.3390/ijerph17113804)
Supplement: Supplementary file 1 [file ijerph-17-03804-s001.pdf]

International Journal of
Environmental Research and Public Health

MDPI

Supplementary Material

Adsorption kinetics and isotherm

Table S1. Kinetic parameters for the Zn(II) adsorption onto Mg-HPA adsorbent.

Pseudo-First-Order Constants			
**Initial Zn(II) Concentration (mg/L)**	**K_1(g/(mg·min))**	**q_e(mg/g)**	**R^2**
10	0.2832	1.3274	0.8729
20	1.3503	3.8586	0.8695
50	1.7492	5.7500	0.8688
Morrist particle intimal diffusion constants			
Initial Zn(II) concentration (mg/L)	K_1(g/(mg·min$^{1/2}$))	C	R^2
10	0.0328	13.807	0.6803
20	0.0953	17.027	0.7976
50	0.2357	23.888	0.7883
Elovich constants			
Initial Zn(II) concentration (mg/L)	α	β	R^2
10	0.2348	13.165	0.9249
20	0.6561	15.292	0.9769
50	1.3631	20.677	0.9783

Table S2. Isotherm parameters for the Zn(II) adsorption onto Mg-HPA adsorbent.

Freundlich Constants			
**Temperature(℃)**	**K_F(L/mg)**	**$1/n$**	**R^2**
25	24.181	0.2147	0.7024
35	26.495	0.2199	0.7528
45	27.404	0.2374	0.8476

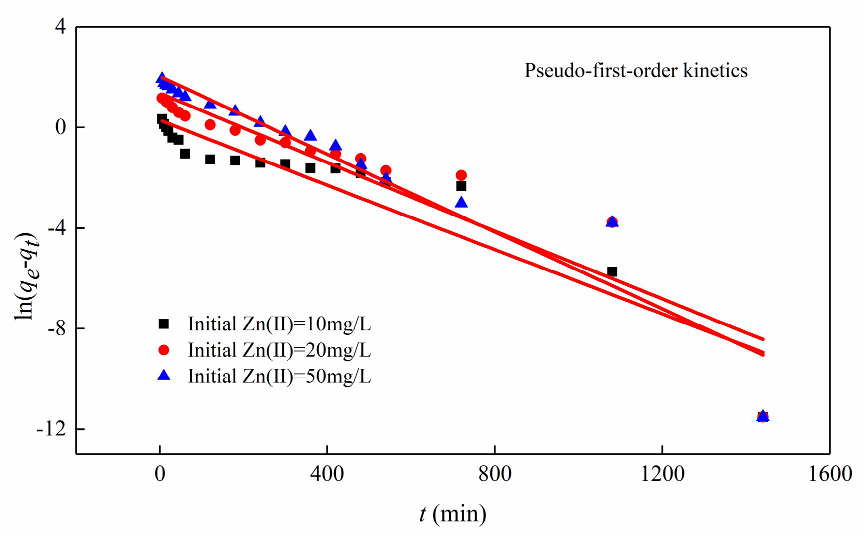

Figure S1. Pseudo-first-order kinetics for Zn(II) adsorption onto the Mg-HAP adsorbent (initial pH=6; initial concentration 10,20, and 50 mg/L; adsorbent dose 0.25 g/50 mL; 25 °C).

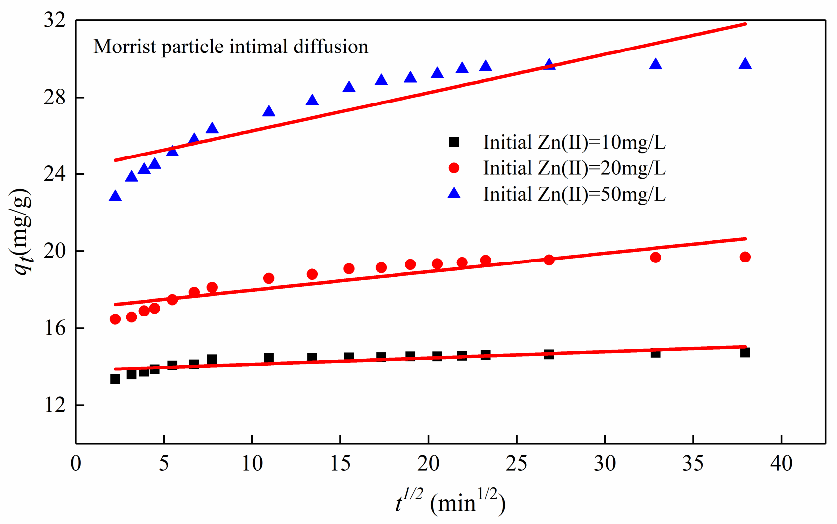

Figure S2. Morrist particle intimal diffusion model for Zn(II) adsorption onto the Mg-HAP adsorbent (initial pH=6; initial concentration 10,20, and 50 mg/L; adsorbent dose 0.25 g/50 mL; 25 °C).

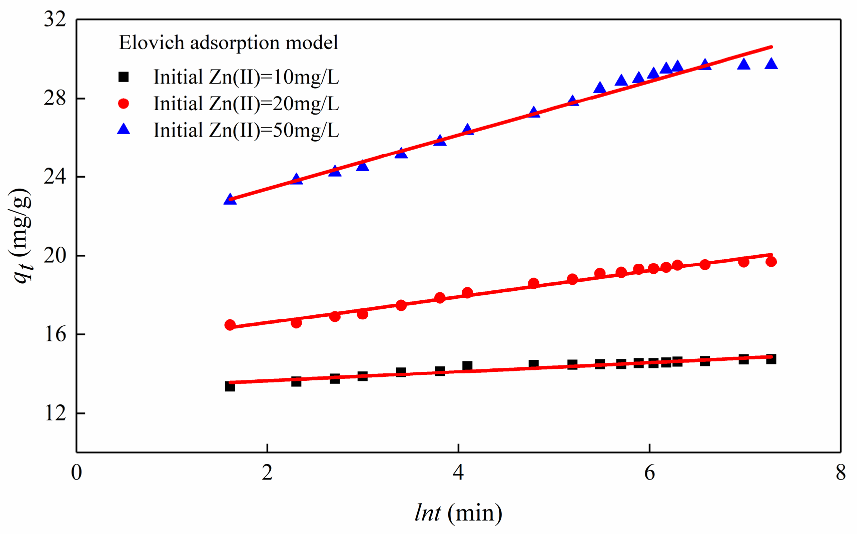

Figure S3. Elovich adsorption model for Zn(II) adsorption onto the Mg-HAP adsorbent (initial pH=6; initial concentration 10,20, and 50 mg/L; adsorbent dose 0.25 g/50 mL; 25 °C).

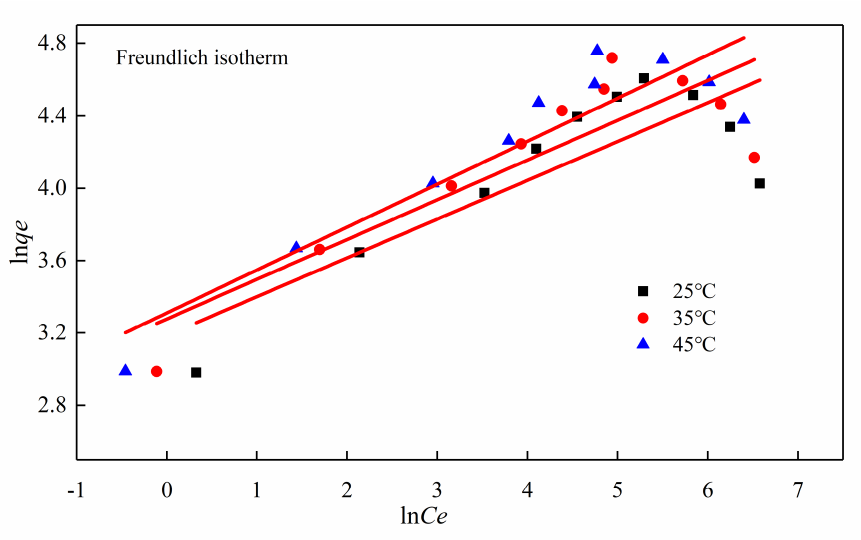

Figure S4. Freundlich isotherm for Zn(II) adsorption onto the Mg-HAP at 25°C,35°C and 45°C(initial pH=6; initial concentration 10,20, and 50 mg/L; adsorbent dose 0.25 g/50 mL).

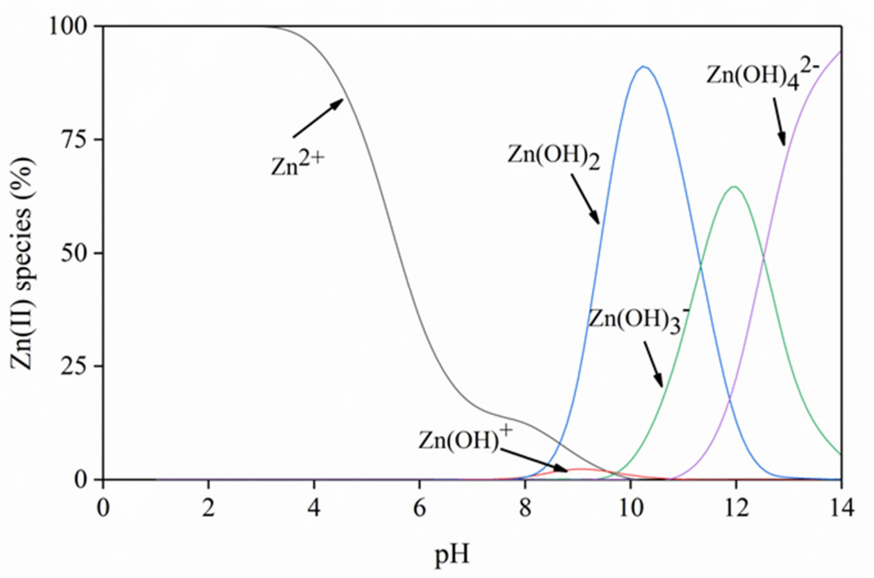

Figure S5. Various forms of zinc in aqueous liquor.
